# A Just Assemblage in Mental Health Services—the Necessity of and Possibilities for Service Diversity

**DOI:** 10.3389/fpsyg.2021.725385

**Published:** 2021-08-10

**Authors:** Rolf Sundet

**Affiliations:** Department of Health, Social and Welfare Studies, Faculty of Health and Social Sciences, Center for Mental Health and Substance Abuse, University of South-Eastern Norway, Drammen, Norway

**Keywords:** universality, dismantling of the welfare state, mental health, assemblage, justice

## Abstract

Basic to the Norwegian welfare state is the principle of universality; every citizen has the right to equal health care and social services. Experiences from a family team in mental health care for children and adolescents exemplify challenges for mental health work in this welfare state. These experiences indicate an ongoing process of dismantling the welfare state, disguised as managerial changes and reorganizations. The argument is put forth that these changes and reorganizations that are claimed to benefit service users actually have negative consequences for many of them. These negative consequences are related to how psychological research on and knowledge of mental health and treatment are combined with organizational principles. The concept of an assemblage is introduced as a manner of describing the dominating relationships in health care between the medical model, the randomized controlled trial and new public management in order to provide responsible health care practices. Rooted in a supposition that how we look upon, describe and understand causation defines our views of science in general, how to produce knowledge, what kind of scientific knowledge is most productive, and how it should be implemented in practice, an alternative assemblage is suggested that better realizes the principle of universality. Here justice is about equal opportunities and equal rights to treatments and sets of practices that fit people as unique individuals. Such an assemblage would bring together diverse models of mental health care, a network of multiple research-based knowledges, and service organizations that include the differences and diversity of the population.

## Introduction

For people growing up and living in a Nordic welfare state, a more and less commonly accepted idea and world view is the right of everybody to justice, liberty, and not only the pursuit of happiness, but also its realization. This is further seen as a collective endeavor where the state has a particular responsibility for ensuring that this is made possible for everybody (Vike, 2017). In some countries it might seem naive, but the Nordic mindset of the general population toward the state is that it is benevolent and that it takes its responsibility seriously in terms of ensuring justice, equality, and empowerment of all citizens. It is basically a trusting relationship. No Norwegian politician would seriously argue for the dismantling of the welfare state. It would probably be political suicide to do so. At the same time we see that there are strong arguments that the welfare state needs to change, particularly in order to address national and international economic realities. Within public health and social services we see changes that can be interpreted as an ongoing process of dismantling the welfare state, disguised as managerial changes, and reorganizations (Vike, 2017).

In the following it will be argued that these changes and reorganizations that are claimed to benefit service users (Helsedirektoratet, 2018), actually have negative consequences for many of them. These consequences may be related to how psychological research on and knowledge of mental health and treatment are combined with organizational principles. This may constrain the ability of the welfare state to accommodate the need for diversity of mental health practices. Paradoxically, then, strategies and organizational changes based on psychological research and knowledge that are meant to increase the capacity of the welfare state to preserve justice and equal care opportunities, have the opposite effect for many.

Basic to the welfare state is the principle of universality; every citizen has the right to equal health care and social services; rich or poor, healthy or not, employed or not, where neither financial situation, gender, class, ethnicity nor sexual orientation should make a difference to this universality (Vike, 2017). This article does not argue that this is fully realized in the Nordic countries. Inequalities, injustices, and differences in opportunities exist, but it will be argued that universality is a fundamental principle of the welfare state that it continuously seeks to realize with more or less success. The concern of this article is that the organizational changes and reorganizations we can observe in our health care system are actually undermining this principle of universality. In mental health this is about how knowledge and its implementation are governed by certain organizational principles; here, knowledge can be seen as a detrimental process attacking the principle of universality. This will be argued by using the concept of *assemblage* (Deleuze and Guattari, 1988; DeLanda, 2006, 2011) as a descriptive device for explaining this situation.

Since we are here concerned with the possible effects of organizational changes and procedures on treatment practices, we need to consider the concept of causation. It will be argued that an important part of the problem is how causation is conceptualized and the consequences drawn from this conceptualization concerning how to view science and research and how one seeks to implement the generated knowledge. Finally, one's view of the individual must be related to how causation is conceptualized and understood. Conclusions will be presented on how possible detrimental effects can be reduced and hopefully eliminated.

## From System to Assemblage

The experiences of practical therapeutic work that form the experiential basis for the arguments and perspectives of this article are from work in a family team in mental health care for children and adolescents in a Norwegian city (Sundet, 2009, 2011). This team had as its inspirational “universe” the trajectory of theoretical ideas that has its origin in systems theory and especially the work of Bateson (1973) with all the diverse lines of development that this tradition has ended up with (Lorås et al., 2017). The dictum of systems theory is that “…the whole is more than the sum of its parts” (Bertalanffy, 1968). It is not about the individual parts of a system, but about the relationships between the parts, e.g., the members of a family (Rivett and Street, 2009). The key idea is the centrality of the relationships between persons and not individual characteristics of persons. It is the relationships between the parts that make the whole something more than the sum of the parts and when parts are related what arises can be named as “emergent” (Bertalanffy, 1968).

It can be argued, at least in the Norwegian context, that the focus has changed from emergent phenomena toward the content of relationships. The introduction of developmental psychology into family therapy (Hansen et al., 1994; Johnsen et al., 2004; Dallos, 2007) has led to more and more studies of the quality of relationships and what are developmentally sound and healthy relationships, and what lead to developmental problems that may explain psychopathology and mental challenges. The key concept is attachment (Groh et al., 2017). Grounded in the work of Bowlby (1969), attachment is seen as inherent biological motivation toward seeking protection from a caregiver when confronted with or experiencing danger. How the child reacts under such conditions can be divided into two main attachment patterns, secure, and insecure, with the latter again divided into avoidant, ambivalent/anxious and severe/disorganized (Dallos, 2007; White and Gibson, 2019).

From the point of view of this author, developments in family therapy and more generally in mental health for children and adolescents, as a combination of system orientation and an increasing focus on attachment and the emotionality of the caregiver-child relationship, have resulted in both a strengthened and a restricted focus in family therapy. The focus on quality, especially the emotional quality of the relationship between child and caregiver, has been strengthened. Further, attention to possible detrimental and traumatic content in this relationship has increased (Dallos and Vetere, 2009; Groh et al., 2017). One consequence of this is that in Norway we see a proliferation of psycho educationally oriented programs such as Circle of Security Parenting (Cooper et al., 2005) and Parent Management Training (Patterson and Oregon, 1982) aiming to advise parents on how best to solve developmental challenges in bringing up and caring for their children. This article does not deny the helpfulness of these programs for many parents, but the increase in these forms of practice limits diversity of practices. One example of this is how attachment theory has certainly led to a strengthening of the diagnostic gaze and practices in providing support to children, adolescents, and their families (White and Gibson, 2019). Experiences from family therapy practice (Sundet, 2011) are that the needs and challenges of many families go far beyond problematic emotional relationships between child and caregiver.

Let me introduce some brief examples. In one family with very limited financial resources a main therapeutic task was to obtain enough funds from the social services to provide the son with the equipment needed to snowboard with his friends. In another family the mother struggled with bodily pain. She was diagnosed with somatization, which she rejected. In the referral to our team, it was asked whether this rejection was due to her being resistant and in denial of a psychological etiology of her problems. Siding with the mother, the therapist started a campaign to have the diagnosis changed. Over time this succeeded. Parallel to this, a very pleasant and interesting collaborative relationship arose between the mother, the rest of the family, and the therapists. As she was referred to a pain clinic which managed to offer some relief to her pain, the family therapy instead developed into an attempt to create a more enjoyable life with less focus on pain and its etiology. Working with a family with very poor housing, the therapist's task was to help improve their home. The Norwegian word “dugnad” describes a form of voluntary collaborative work where every participant is seen in a symmetrical role of equality. This became a meaningful description for all parties and the word “therapy” moved into the background. Former psychiatric symptoms of depression and anxiety in the parents and children were reduced in this joint collaborative process where tidying, painting, and work on the aesthetics and practicalities of creating a pleasant home was the main concern. The final example shows how texts and information material from social media like Facebook created anxiety and fear of losing their children in a family which was placed under the supervision of the child protection services. Instead of attending to the primary concern of the child protection service, a reading, and discussion group was set up to explore what elicited the anxieties of the family. Different perspectives on parenting were discussed. Through reading texts, the family gradually put words to how they felt and what they considered good parenting. Indirectly this also answered many of the concerns of the child protection services. The interesting part was that this change did not arise through talking to professionals from the child protection services, but by engaging with the texts from social media. Interacting with the texts became more important than interacting with people, but it had an effect on how the parents and children acted together, which again addressed the concerns of the child protection services.

In all of these examples, people are certainly part of what was implemented, but it includes more than the people and their relationships. There were emotional interchanges, both regulatory and invigorating, and also challenging situations, but there was more. There was the accessibility of material objects (snowboards and clothes); there was rebellious and non-cooperative behavior toward the diagnostic system and its meanings; there was the doing of actual manual work in changing the aesthetics of a house, and lastly, there was interplay with texts more than with people. This requires a concept that includes but also moves us beyond people and their relationships.

### The Assemblage

The origins of the concept of assemblage are found in the work of Deleuze and Guattari: “We call an assemblage every constellation of singularities and traits deduced from the flow…” (Deleuze and Guattari, 1988). Assemblages are emergent wholes that are products of historical processes. They are both irreducible and decomposable and are defined by their properties, tendencies, and capacities. Assemblages are universal singularities and as such historically unique, but they always belong to more or less similar assemblages (DeLanda, 2011). Like the concept of system, an assemblage is more than the sum of its parts. One major difference is that with an assemblage it is explicitly stated that the related parts can be objects, texts, signs, persons, meanings, animals, feelings and emotions, discourses, and semiotic systems (Stivale, 2014). There is no limit to what can be related within an assemblage because here, as in systems, it is the relations that are central.

“An assemblage is not a set of predetermined parts (such as the pieces of a plastic model airplane) that are then put together in order or into an already conceived structure (the model airplane). Nor is an assemblage a random collection of things, since there is a sense that an assemblage is a whole of some sort that expresses some identity and claims a territory” (Wise, 2005).

In everyday family therapy practice I have often been confronted with the fluid and changing nature of the contemporary family. A 9 year old boy expressed worry about how my childhood had been when he discovered that I only had four grandparents. He had eight. He asked me about Christmas and stated that with eight grandparents you were sure to get a good number of presents. He had the experience, just before his parents' divorce, then having only four grandparents, that the situation concerning Christmas presents had become precarious. With the combination of a low number of grandparents and high conflict between parents that also involved the grandparents, his wishes for Christmas had got lost in the turmoil of divorce. His strong argument was that an increase in the quantity of grandparents, due to his parents having established new relationships, would also improve the Christmas present situation. He admitted that it could be a bit stressful to keep up a relationship with all eight, and that he was now used to changes both in these relationships and how close or distant he felt toward them, but still this had had no effect on the Christmas situation, nor on his birthday for that matter.

It was a convincing argument for the opportunities and possibilities that the contemporary, fluid, and changing family had for him. It was also an educational moment for the present author, who then realized that the quality of relationships is not everything. The number of people and possible combinations of these relationships seemed to maintain a secure and predictable situation for this boy regarding material goods at Christmas and birthdays. Implicitly, personal relationships also followed from the presents (Mauss, 2002). The system concept and its practical designs and forms, especially with the centralization of attachment, the emotional quality of relationships and appropriate responsiveness, does not do justice to the diverse, fluid, changing, and heterogeneous aspects of contemporary life that impact families and their members. The assemblage “…is not the arrangement or organization but the process of arranging, organizing, fitting together” (Wise, 2005). There are heterogeneous elements in this that interrelate, continuously in flux and flow. In principle anything can be included in an assemblage, then to be excluded, left, and switched with other elements and relations. Elements and their relationships are not, as stated, limited to persons. Any element, object, feeling, expression, sign, aspects of the environment, and geography can be included, and then affect and be affected by other elements and relations.

## The Medical Model, the Randomized Controlled Trial, and New Public Management: An Assemblage of Help and Exclusion

Experiences of working in the family team during the first 10 years of the new millennium became more and more marked by intrusion. We felt that we were under surveillance, and consequently became more and more afraid of making mistakes. Suddenly the metaphor of Bentham's Panopticon (Foucault, 1977) became a fitting description for our experience and situation. A specific manifestation of this is how we started to change the way we wrote in medical records. Almost imperceptibly our writing became less oriented toward being a tool for communicating our practice and understanding of the family and our work to colleagues, and more and more concerned with protective maneuvers against possible legal and judicial questions and accusations connected to our work. The space for discussions and reflections on mistakes as opportunities for learning was reduced. Instead structured procedures became authoritative paths for avoiding mistakes, while pressure also increased to use manualized forms of therapy. The battle call was “follow the guidelines” with the almost religious belief that this would eradicate mistakes and create effective and helpful treatment for our service users. Our politicians, the public health bureaucracy, and the professional associations of health, and social workers all took part in strengthening this pressure. This was done by emphasizing two ideology-based institutional models, the medical model (MM) and the use of randomized controlled trials (RCTs), which resulted in specific procedures in our daily practice.

### The Medical Model

The MM contains three tasks organized sequentially: assessment, diagnosis and treatment. In accordance with the concept of evidence-based practice, the American Psychological Association has published a declaration on what is best evidence-based psychological practice (Force, 2006). This includes the best available research-based knowledge, the experience of the clinician and the preferences of the service user. Here, RCTs have a privileged position as a basis for determining best practices (Chambless and Hollon, 1998).

It is somewhat ironic that the MM is chosen as the best way to ensure effective and helpful practices since it does not explain the empirical findings of psychotherapy research. According to Wampold and Imel (2015), this model does not have empirical support. To make the best sense of research data, they chose what they called the contextual model. Their analysis of different meta-analyses of psychotherapeutic practices reveals no support for a strong relationship between diagnosis and outcome in psychotherapy (Wampold and Imel, 2015). In addition, in preparing this text, it has been difficult to find research that confirms an efficacious relationship between assessment and outcome. Instead of evidence-based practice, their solution to ensuring helpful practice is practice-based evidence “…which uses data about progress of clients in practice to improve the quality of care” (Wampold and Imel, 2015). This underlines the importance of routine outcome monitoring (Tilden and Wampold, 2017), i.e., the use of feedback on process and outcome from the service user to the therapists. This leads to a focus on the relationship between the individual and what is efficient and helpful in that person's life.

### The Randomized Controlled Trial

The aim of an RCT is to show that when persons with a specific diagnosis are exposed to a theory-specific method, there is a connection between the method used and a measurement of change or outcome. An RCT thus provides knowledge of efficacy at the group level (Anjum, 2016). Efficacy and outcome refer to the (diagnostic) group investigated. The transformation of this group level knowledge into a helpful practice for the individual depends upon a certain theoretical rationale. Kennair et al. (2002) write: “There are variations between humans, but there also is a relatively uniform human nature. This means that investigations that work on large groups of humans will probably work for random individuals” (Kennair et al., 2002).

The aim of this paper is not to dispute the importance of all those aspects of the human species and life that we share with each other. Instead we would argue that these aspects of a possible common nature are expressed in a diverse and heterogeneous manner in each of us, and that this demonstrates the uniqueness of each individual. To use Hanne Arendt's words:

“Plurality is the condition of human action because we are all the same, that is, human, in such a way that nobody is ever the same as anyone else who ever lived, lives or will live” (Arendt, 1958/1998).

The challenge with RCT-based knowledge is that it can easily lead to a view of those that do not fit the general findings as random examples or as statistical outliers, with the risk of excluding them from treatment. A more productive conclusion would be that their status as outliers indicates methodological challenges. Such a conclusion would be an impetus for continuously working out how to create new practices that could fit the outlier. This is where our team experienced the emergence and practice of new public management (NPM) as a destructive constraint on our practice and possibilities for being helpful.

### New Public Management

The public sector in Western countries has undergone major transformations and adjustments. These are connected to a neoliberal market-oriented ideology. When put into an organizational practice we often find this under the name of new public management (Ekeland, 2004; Karlsson, 2015; Rustin, 2015). Here questions of health and social care are moved from the political arena to the arena of the market. Theory-specific methods tested with RCTs and found efficacious are qualified for membership in the health service market. The others are easily excluded. Accessibility and management of services takes place through standardizing how one gains access, which method to use and how it should be implemented. Standard service provision, management of accessibility, and documentation of following the standard procedures become central activities of therapists. This reduces the autonomy of the practitioner. Although there is room for adjustments, the emphasis is on standardization through the use of adherence measures (Ekeland, 2004). As stated above, in the Nordic welfare states the legislation prescribes equal opportunity for equivalent services. What our practical therapeutic work has taught us is that in order to provide equivalent services for different people, there must be heterogeneity of methods (Sundet, 2011). Equivalent services mean accessibility of different methods for different clients. RCTs cannot provide us with this because an RCT only gives us knowledge of a group.

A combination of NPM and RCT-based knowledge leads to services for classified individuals, i.e., generalized persons, in Norway individuals classified through an ICD-10 diagnosis. The neoliberal individual becomes a generalized individual stripped of whatever outlier aspects or uniqueness that makes this individual a person. In the company of the medical model with the valorized knowledge from RCTs, paradoxically, NPM becomes an anti-individual endeavor. Personhood shows itself and is realized in relationships with others. Take the person out of relationships, away from natural, material, social and cultural contexts, and we are left with a generalized and reified person, with no possibility of survival, except in texts of theory and research.

### The MM-RCT-NPM Assemblage

As stated above, in an assemblage the parts and processes that are related can be material objects, texts, signs, persons, meanings, animals, feelings and emotions, discourses, and semiotic systems (Stivale, 2014). There is no limit to what can be related within an assemblage because here, as in systems, it is the relations that are central. Further, the elements of the MM, RCT and NPM and their entanglement into an assemblage are in a constant flux of affecting and being affected by each other and all those who come into contact with this assemblage. Although the MM and RCT are both important and valuable in their own respect, something happens when they are coupled with NPM. NPM is a political force, a manner of organizing power relations and ways of managing all the elements of the MM and RCTs. NPM is the actual realization and concrete implementation of an ideology named neoliberalism, whose solution to all questions of living is the market and the competition within it. The market will solve all medical, social and psychological challenges, and problems (Karlsson, 2015).

Although the market orientation ideally aims at freedom of choice, NPM introduces control into the equation when entangled and intertwined with the MM and the use of RCT- generated knowledge. The entanglement of the MM and RCT- based knowledge results in an evidence-based practice in which standardization becomes a main tool. Freedom of choice is thus restricted to such methods. Paradoxically then, the individual's freedom of choice becomes subordinated to both a standardization of which method to use (an evidence-based method) and a standardization of how the chosen method is applied (adhere to the manual). We find governmental statements pointing out that standardization should not overrule specific individualized choices in both the chosen method and how it is applied (Statens helsetilsyn, 2001), but at the same time clinical pathways are now subject to a clear requirement for the use of the MM and evidence-based methods (Helsenorge, 2020). On the one hand, individualization and service user participation are central to these clinical pathways. On the other hand, they are bound to specific diagnoses, where the sequence of assessment, diagnosis, and then treatment is the ruling practice and where “best practices” should be used, i.e., evidence-based methods. Let me illustrate the challenges and tensions in this MM-RCT-NPM assemblage.

Ann, 13 years old, was referred to our team due to having dropped out of school. A talk with her mother and father clearly revealed that she struggled with anxiety in social situations, but especially in the classroom. Ann herself was mostly silent and it became clear that she did not particularly trust therapists. Our team worked with children, adolescents, and families who had tried treatment at the outpatient service, but had not found this helpful. One important question for our team, in the first contact with the family, was to ask if the family had any experiences from prior treatment that could help us find out what we should not do. The spontaneous response of the mother, in an insisting, almost aggressive, tone of voice was the following: “Could we please stop this exposure thing!”

Investigating this response led to a story of how the previous therapist had recommended the use of an evidence-based, systematic exposure training procedure. The first elements that could be included in our assemblage were the assessment process and the diagnosis. Ann and her parents felt that they were listened to. The next element was that the diagnosis was an indication of what kind of treatment should be used. Again Ann and her parents felt that they were being listened to. Something could be done. A third element was the implementation of an evidence-based treatment program. Here RCT makes its appearance as strongly recommending the use of a systematic exposure program that had been tested in such a procedure. A diagnosis and being listened to and given best practice felt reassuring for the family, although Ann had expressed that all this assessment was a hassle, and that it did not help her. That was actually the beginning of her distrust in therapists. The next element, treatment, was started, but then a number of problems arose for both the family and their therapist in the outpatient clinic. Ann felt that the exposure procedures made her feel worse, and she started to protest and finally refused to take part in the program any further. All trust was gone on her part.

In this process the parents were instructed to encourage Ann to attend the treatment sessions and their procedures. This brought Ann and her parents on a collision course and tensions arose. The parents experienced being squeezed between their daughter's growing despair and anger toward them, and increasing pressure from the therapist to “support” the treatment. In this part of the process all the relationships changed from an atmosphere of trust and collaboration to mistrust, anger, and despair. The parents argued with the therapists that there was something wrong with this treatment, while the therapists maintained that it was the best, given evidence from research, which they were obliged to use. The parents argued that it did not suit their daughter. They also found that their refusal brought forth responses from the therapists that they interpreted as an evaluation of their fitness as parents. If they did not manage to provide this support, it would be a sign of bad or inadequate parenting, which again could lead to the family being referred to the local child protection service. This aspect was not communicated by the therapists, but because of the conflicts in the talk between the parents and the therapists, the parents were reminded of the many stories in the daily press, Internet, and TV that focused on how lack of proper parenting could lead to the child protection service taking over the parenting role. Included in this assemblage, then, are elements from public discourses and narratives on poor parenting skills and lack of proper caring, often supported by expert statements rooted in psychological theories on development and parenting. Although maybe just fantasies on the part of the parents, the stories, and arguments from the press and media using expert statements from researchers and therapists could clearly explain why such fantasies arose in the ever deteriorating climate between this family and their therapists. At this point it was decided that Ann and her parents should be referred to our team, whose task was to explore other ways of working that could be helpful.

At one point after our team had started to work with the family, the previous therapists were invited to a meeting in order to examine the family's experiences in more detail. This meeting revealed the intrusion of NPM and how this meshwork (Ingold, 2013) of principles, guidelines, and ideological premises had a powerful grip on both the family and their therapists. The therapists stated that they understood the frustration of the family. They themselves, due to the rules and guidelines on how to implement an evidence-based, effective, economic, and equal service under Norwegian law, had been constrained by being unable to follow the preferences of the family in the destructive situation due to the exposure approach. Pressure to produce structures of assessment and diagnosis with limited choice outside the evidence-based methods that were the market winners of health care methodology had forced them to follow a path that they also saw only increased the problem. Their solution was to refer the family to the family team. This particular team was fortunate in that one of its senior managers in the organization had realized that there were families who did not fit the increasingly standardized procedures for assessment, choice and implementation of the method. There was a kind of tacit acceptance that as long as the team could do something helpful, one could turn a blind eye to the fact that the dominating principles in mental health care of diagnosis and a standard treatment pathway were not being followed. Although there was room for individualized treatment in this standard process, this family had experienced its clear limits.

What happened in this situation was that the team still felt under the spell of the idea that an evidence-based treatment had to be used. What brought us out of that spell was the mother simply stating that we needed to do something else than exposure, and when one of the team members somewhat perplexed asked, “Could we?”, she simply said “Yes”. The rest is a long story with challenges and setbacks, but in the end Ann was back in the classroom. When asked how this came about, she stated, “My desire for my friends became bigger than my anxiety”. What we learned was that when systematic exposure is of no use, bet on desire.

This example could easily be turned into a villain-hero script, with the previous therapists as the villains and our team as the heroes. Our experience suggests a completely different script. If and when we were able to be helpful this was built on what the previous therapists had done. These therapists working at their best within the circumstances and contextual forces of the outpatient clinic made us realize what we should not do. The forces affecting the previous therapists were the demands of the MM, of using evidence-based treatments and working under the strong influence of the idea that when you know something of the many, this also applies to the person(s) and family in front of you (Kennair et al., 2002). Generalized knowledge informs work with individuals and families. Organizational and managerial practices based on NPM stress standardization, adherence to guidelines, and a digital system of documenting assessment, diagnosis, and treatment, which are seen as a temporal sequence on which to base work organization and the length of time service providers are in contact with clients.

However, the family team found that breaking these rules and regulations opened up opportunities and possibilities. The main point was no longer these guidelines and regulations, but to establish a collaborative relationship where the preferences and suggestions of the families became the central focus of our work (Sundet, 2011; Sundet et al., 2020a,b). From this point therapy was created in cooperation with the family. The script was now stories about how we were standing on the shoulders of our colleagues. Without them we could not have done what we were doing, and our dependency on seeing what they had done enabled alternatives. Formulating the MM-RCT-NPM assemblage became a blueprint for creating strategies and actions of resistance toward the dominating practices resulting from this assemblage. For us, the most liberating assumption was that in reality what happens is not best described and understood by the idea that what concerns the many also goes for the individual. Instead the uniqueness of the single person, within the person's relationships with others, became a necessary starting point and guiding idea for our work. This led to the realization that the concept of causation as brought forth through the use of RCTs, and which was explicit or implicit in this assemblage, had to be changed.

The concept of an assemblage, then, is an invitation to consider all these different aspects that can be related and reciprocally affect everything and everybody that comes into contact with or is included in the assemblage. It must also be emphasized that when everybody and everything in an assemblage can be in a relationship of mutual influence, this implies statements about causation. The concept of causation that is operational in mental health care cannot do justice to the fluid, heterogeneous, and complex nature of an assemblage. We therefore need to focus on an alternative formulation of causation. With this in place we can start to view the situation and predicament of mental health care from the perspective of an assemblage, namely the MM-RCT-NPM assemblage that has grown on us and gained strong momentum since the late eighties. This assemblage is experienced by the family team as a dominating political, discursive, and ideological force that defines the professional and organizational reality of health care workers; however, let us first consider causation.

## Causation

Why causation? The supposition of this paper is that how we look upon, describe, and understand causation defines our views of science in general, how to produce knowledge, what kind of scientific knowledge is most productive, and how it should be implemented in practice. A crucial element in our emerging understanding of the MM-RCT-NPM assemblage was an appreciation of the assumptions about causality that are embedded within it and provide its intellectual force.

Our experience from working within the MM-RCT-NPM assemblage is that it relies on an understanding of causation connected to RCT-based knowledge. Anjum (2016) identifies this as similar to the ideas of the Scottish philosopher David Hume, formulated in three central principles: time asymmetry (the cause always precedes the effect), contiguity (the effect is temporally close to the cause), and constant conjunction (the association is repeatedly and constantly observed) (Hume, 1978/1739). From such a perspective, causation becomes something that is identified in statistical patterns and frequencies of aggregated data. The processes of the MM-RCT-NPM assemblage, then, depend on this specific perspective on causation, which necessitates standardization as the overall manner of ensuring implementation in line with its research findings. The therapeutic practices studied in the RCT must be replicated in practice. Standardization enables the repetition of the same in the practical context. The ideas on causation that the RCT builds on have this repetition of sameness as their basis. The same or the similar is aggregated, and outliers as differences are either removed through randomization or canceled out. The frequency and statistical pattern found becomes what must be replicated in practice, which warrants the use of manuals to be followed when the findings of an RCT are to be realized in actual therapeutic work. Although live therapeutic work must be adapted to the client, this adaptation must also retain the basic pattern of practices that are productive and effective, based on the RCT. There is nothing wrong or dangerous in itself in this practice, but when coupled to organizational steering of practices in line with ideological, political and economic goals, and constraints, this increases the risk of marginalization and exclusion of those who do not fit the pattern. Further, the autonomy of the practitioner and the clients in choosing care and treatment is constrained by these goals and steering. In the health care system this is realized by standardizing the procedures to be followed in practical work. This is the medical model with its sequence of assessment, diagnostics, treatment planning and implementation. Reduction of the diversity and flexibility of therapeutic work becomes the core of an unjust assemblage. A just assemblage must be able to include outliers and those who differ from what is found helpful in an RCT. This raises the need for an alternative theory of causation that goes beyond frequencies and that can include the individual.

### Dispositionalism

In the frequency theory of causation there are only two ways to understand what is happening, either necessity or contingency and possibility (Anjum and Mumford, 2018). Dispositionalism (Mumford and Anjum, 2011) takes the middle ground concerning necessity and contingency. The middle ground is for Anjum and Mumford (2018) a situation where there is no necessity, but still some effects are more likely than others. Further, an effect arises from the interplay and interaction of several causes, each of which alone does not give this specific effect. For these philosophers causes are dispositions.

Mumford and Anjum (2011) suggest an ontology where causes are genuinely connected and not only joined through correlations in aggregated data. Their explication of causation goes beyond the Humean principles stated above. In their ontology causes do not necessitate effects. Instead, causes tend to have effects. Causes are dispositions and tendencies. The core idea of dispositional ontology is that real causal powers exist and they can “…contribute to or counteract a certain effect” (Anjum, 2016). This paves the way for science and research-based knowledge that goes beyond the group level frequencies and generalizations found through the use of RCTs. Science becomes something more than aggregating data. It is about suggesting causative mechanisms for observed and reported effects. Causes are not found in perfect regularities. It brings science beyond the identification of regularities on a group level and into understanding “…the ‘why’ and the ‘how’ in its emphasis on understanding, meaning, and sources” (Anjum, 2016). Two aspects of this ontology are especially important for the arguments in this text. First of all, dispositionalism implies that there can be a cause that happens only once under the sky of eternity. This is “…consistent with causal singularism…” that “…causation happens in the particular case without an assumption of a corresponding causal law…” (Anjum, 2016). The singular, unique, and once appearing act, event, factor or situation must be included as a possibility in any research or practical situation. Secondly, this view of causation underlines that causes are context-sensitive in the sense that the slightest change in contextual parameters and aspects will determine whether causation appears. Causes as dispositions act together in producing certain effects. One way of stating this is that dispositions have manifestation partners that produce an effect when acting together (Anjum, 2020). Causation then implies both singularity and complexity. This view of causation makes us aware that any generalization must, in the practical context of health care work, be checked to determine whether it fits with the actual person or people that the knowledge is supposed to apply to. This is the true sense of an individualized health care practice.

## A Just Assemblage in Mental Health

Under the dominance of a frequency theory of causation, intertwined and entangled with the MM in organizational practices based on control and standardized procedures, it will be difficult to include the outliers of an RCT. In an RCT the outliers might be equalized and rendered unimportant, but they come through the door of the office or the ward as actual people. Therefore we need an assemblage that can include uniqueness, the individual person and the diversity and variety that a mental health care system must expect to meet. Based on Ashby (1958) law of requisite variety, we know that in order to address variety, we need variety. In order to address the variety both within a diagnostic group and between diagnostic groups, or using any other manner of describing the population of people with mental health issues, we need the same range of variability and diversity in our ways of working with these people in order to be helpful. The key to realizing a just and equal mental health care system is the development of an organizational structure that not only offers diversity in mental health care practices but can also develop new practices that can be helpful for clients when we are confronted with something that none of us has ever met before.

Justice is about equal opportunities and equal rights to treatments and sets of practices that fit each of us as unique individuals. Justice, then, is about providing care outside a tyranny of generalizations, of not seeing clients as one of many, as a generalized object fit for a treatment opposed to uniqueness. Further, when a client falls outside the parameters of the generalization, this must not be taken as not being accessible for treatment, being unmotivated or having some kind of deficiency that explains why treatment is not helpful. It requires an assemblage that is willing to see lack of change or help as a methodological challenge for researchers and health care workers and not as a defect of the person in need of help. Such an assemblage will be built around the idea that change and becoming are continuous states, always presenting new challenges, where uncertainties and dilemmas rooted in differences are a necessary part of the assemblage.

In such an assemblage the MM is only one of many possible models that can be used as tools in identification and treatment of mental health problems and challenges. From psychotherapy research another option would be the contextual model argued for by Wampold and Imel (2015). Models that are more service user and collaboratively oriented and built around service user feedback would also be candidates (Sundet, 2011; Sundet et al., 2020b). It is beyond the scope of this paper to outline such models in detail. The point is that the model(s) used must be able to incorporate the diversity that follows from a view of people as unique and of causes as dispositions. Knowledge in such an assemblage must go beyond the production of generalizations and include knowledge of single cases, and of mechanisms of change as possible unique events. This means establishing a knowledge policy that does not establish knowledge hierarchies, but sees research methods as part of a network of methods that are created to answer different research questions. In addition, because unfamiliar phenomena and events might arise in people's lives, the treatment situation must be prepared for meeting something that we do not know how to deal with. In such knowledge situations, standardizations, guidelines and patient pathways become suggestions for practice. They are reservoirs of possible actions to choose from, where our choice is always dependent upon the response of the person(s) in the family one is working with. Autonomy for the individual health care worker to choose how to act becomes a necessary part of the assemblage, coupled with the acknowledgment that we are always dependent upon and constrained by the response of the other. Autonomy and dependency are not in opposition in a just assemblage, but conditions for helping clients.

From the perspective argued for here, NPM is an organizational and managerial realization of a political ideology, namely neoliberalism. Politics can be seen as “…those relationships and activities that reflect power and value differences and which influence critical decisions about the distribution of resources, rights, access, opportunities and status” (Reisch and Jayshree, 2012). The elements allocated and constrained in terms of resources, rights, access, opportunities, and status within a neoliberal ideology are chosen through market competition. At the same time, within the MM-RCT-NPM assemblage, only those methods and practices that are theory-specific and standardized through manuals or principles to be followed are included in the market. Since psychotherapy research finds it difficult to differentiate between the effects of different theory-specific methods (Wampold and Imel, 2015), we may ask whether this actually indicates a situation where health care practice becomes a very restricted domain with a low possibility of meeting the actual diversity of needs of mental health care patients in the population.

What would be a necessary condition for the creation of a just assemblage? Diversity and variety imply difference. Diversity is aggregated differences. This means that everybody involved in the just mental health care assemblage must respond to difference in an affirming and inclusive manner. Since part of this condition is that everybody will meet something that is quite unfamiliar, nobody, neither the health care worker, nor the managers and bureaucrats, nor the general public, nor politicians, can escape the necessity of meeting and responding to difference, such as difference in need, ability, background and other aspects. The necessary condition for a just assemblage is the responsibility for responding to and caring about difference, which includes being ready for uncertainty, ambivalence and unpredictability in the situation at hand. It means that whatever happens, nobody escapes choice, choice in how to act in relation to the other(s), who will always represent some kind of difference. In the words of Zygmont Bauman: “Responsibility for choice is …a lonely matter – it rests fairly and squarely on the individual‘s shoulders, as do the consequences of choosing…” (Bauman, 1995).

## Closing Words

A just assemblage in mental health care practice will replace the MM-RCT-NPM assemblage with an MDM-MRK-OID assemblage, namely one consisting of multiple diverse models, multiple research-based knowledges, and organizations that include diversity. MDM means that service users and therapists must be allowed to organize the practical work and its content in various ways. In the family team this meant the right to start with treatment and to let assessment and naming the problem be included as aspects of the treatment and not organized into three different processes as the MM indicates. MRK means to abolish a hierarchy of methods and instead consider the relationships between research methods and the generated knowledge rhizomatically as a network (Deleuze and Guattari, 1988). Finally, the organizational and managerial context of the therapeutic work must allow for the autonomy of the professional on how to act, only constrained by the response of the service user. In the end this creates greater trust in professionals to make choices on how to work and act than afforded by the MM-RCT-NPM assemblage.

A just assemblage affirms on the one hand that no one can escape the responsibility of choosing how to act. On the other hand this responsibility is mutual, which again affirms a collaborative relationship between everybody and everything included. Relationships involving competition, opposition and conflicts must be subordinated to relationships of collaboration (Wilden, 1987), and must always be accompanied by awareness that any difference can easily be transformed into inequality and injustice (Sundet, 2001). The price for freedom of choice is the personal responsibility we all have for taking in, reflecting upon and changing negative consequences of our actions. This means establishing a culture within mental health services that views mistakes and negative consequences as an opportunity for change and for finding more helpful responses to the one in front of us who needs our help. Our feeling of being alone, when confronted with choices of how to live and act, has as its main comfort the fact that this is a mutual predicament for all of us and that we all are in this together. The experience of the family team is that due to being part of a team our aloneness does not imply loneliness. Aloneness can be shared. Collaborative teamwork is a vital ingredient of a just assemblage. The MM-RCT-NPM assemblage, on the other hand, exchanges aloneness and responsibility for choice with the “certainty” established by standard rules, regulations, guidelines, and patient pathways. Freedom becomes freedom from choice and responsibility and although it cannot be denied that there are many who are helped within this assemblage, the real risk is twofold. Firstly, those who do not fit with the recommended methods are excluded, and even worse, have aspects of their functioning ascribed as the cause of the refusal of help. Secondly, and maybe even more unfortunate from the perspective of a just society, is the tendency for responsibility and freedom of choice to be sacrificed on the altar of sameness.

## Data Availability Statement

The original contributions presented in the study are included in the article/supplementary material, further inquiries can be directed to the corresponding author.

## Author Contributions

The author confirms being the sole contributor of this work and has approved it for publication.

## Conflict of Interest

The author declares that the research was conducted in the absence of any commercial or financial relationships that could be construed as a potential conflict of interest.

## Publisher's Note

All claims expressed in this article are solely those of the authors and do not necessarily represent those of their affiliated organizations, or those of the publisher, the editors and the reviewers. Any product that may be evaluated in this article, or claim that may be made by its manufacturer, is not guaranteed or endorsed by the publisher.

## References

[B1] Anjum R. L. (2016). Evidence based or person centered? an ontological debate. Euro. J. Pers. Center. Healthc. 4:2. 10.5750/ejpch.v4i2.1152

[B2] Anjum R. L. (2020). “Dispositions and the unique patient,” in Rethinking Causality, Complexity and Evidence for the Unique Patient. A CauseHealth resource for Healthcare Professionals and the Clinical Encounter, ed. Anjum S.C.W.R. R. L. (Cham, Switzerland: Springer Open), 13–13.

[B3] Anjum R. L. Mumford S. (2018). What Tends to be. The Philosophy of Dispositional Modality. London and New York: Routledge.

[B4] Arendt H. (1958/1998). The Human Condition. Chicago: The University of Chicago Press.

[B5] Ashby W. R. (1958). Requisite variety and its implications for control of complex systems. Cybernetica 7, 83–99.

[B6] Bateson G. (1973). Steps to an Ecology of Mind. Paladin: St. Albans.

[B7] Bauman Z. (1995). Life in Fragments. Essays in Postmodern Morality. Oxford: Blackwell.

[B8] Bertalanffy LV. (1968). General Systems Theory. Foundations, Development, Applications. New York, NY: George Brazillier.

[B9] Bowlby J. (1969). Attachment: Attachment and Loss: Vol.1: Loss. New York, NY: Basic books.

[B10] Chambless D. L. Hollon S. D. (1998). Defining empirically supported therapies. J. Consult. Clinic. Psychol. 66:7. 10.1037/0022-006X.66.1.7 9489259

[B11] Cooper G. Hoffman K. Powell B. Marvin R. (2005). “The circle of security intervention: differential diagnosis and differential treatment,” in Enhancing early attachments: Theory, research and policy, eds. Berlin L.J. Amaya-Jackson L. Greenberg M. T. (New York, NMY: Guilford), 127–151.

[B12] Dallos R. (2007). Attachment Narrative Therapy. Integrating Narrative,Systemic and Attachment Therapies. Maidenhead: Open University Press

[B13] Dallos R. Vetere A. (2009). Systemic Therapy and Attachment Narratives: Applications in a Range of Clinical Settings. London: Routledge. 10.4324/9780203881453

[B14] DeLanda M. (2006). A New Philosophy of Society. Assemblage Theory and Social Complexity. New York, NY: Continuum.

[B15] DeLanda M. (2011). Philosophy and Simulation. The Emergence of Synthetic Reason. New York, NY: Continuum.

[B16] Deleuze G. Guattari F. (1988). A Thousand Plateaus. Capitalism and Schizophrenia. London: The Athlone Press.

[B17] Ekeland T.-J. (2004). Autonomi og evidensbasert praksis.

[B18] Force A. (2006). APA presidential task force on evidence based practice. Am. Psychol. 61, 271–285. 10.1037/0003-066X.61.4.271 16719673

[B19] Foucault M. (1977). Overvåkning og straff. Det moderne fengselssystems fremvekst. Oslo: Gyldendal.

[B20] Groh A. M. Fearon R. P. van IJzendoorn M. H. Bakermans-Kranenburg M. J. Roisman G. I. (2017). Attachment in the early life course: meta-analytic evidence for its role in socioemotional development. Child Develop. Perspect. 11, 70–76. 10.1111/cdep.12213

[B21] Hansen B. Johnsen A. Sundet R. (1994). Daniel stern og familieterapi: implikasjoner av nyere utviklingsteori for familieterapi. Fokus p familien 22, 94–108.

[B22] Helsedirektoratet (2018). Nasjonal plan for implementering av pakkeforløp for psykisk helse og rus 2018–2020 (IS-2734).)

[B23] Helsenorge (2020). Hva er pakkeforløp for psykisk helse og rus? [Online]. Available online at: https://www.helsenorge.no/psykisk-helse/pakkeforlop-for-psykisk-helse-og-rus/hva-er-pakkeforlop-for-psykisk-helse-og-rus/

[B24] Hume D. (1978/1739). A Treatise of Human Nature. Oxford: Clarendon Press.

[B25] Ingold T. (2013). Making. Anthropology, Archeology, Art and Architecture. London and New York: Routledge.

[B26] Johnsen A. Sundet R. Torsteinsson V. W. (2004). Self in Relationships: Perspectives on Family Therapy from Developmental Psychology. London and New York: Karnac.

[B27] Karlsson B. (2015). Markedsliberalistiske forskyvninger i det psykiske helsefeltet–om forholdet mellom politisk styring og faglig disiplinering. Nordisk tidsskrift for helseforskning 11, 153–162. 10.7557/14.3719

[B28] Kennair L. E. Aarre T. F. Kennair T. W. Bugge P. (2002). Evidence-based mental health—the scientific foundation of clinical psychology and psychiatry. Sci. J. Sci. Health Policy 3, 196–209.

[B29] Lorås L. Bertrando P. Ness O. (2017). Researching systemic therapy history: in search of a definition. J. Fam. Psychother. 28, 134–149. 10.1080/08975353.2017.1285656

[B30] Mauss M. (2002). The Gift: The Form and Reason for Exchange in Archaic Societies. Routledge.

[B31] Mumford S. Anjum R. L. (2011). Getting Causes from Powers. Oxford: Oxford University Press.

[B32] Patterson G. R. Oregon E. (1982). A social learning approach, Volume 3: Coercive family process (Eugene, OR: Castalia).

[B33] Reisch M. Jayshree S. J. (2012). The new politics of social work practice: understanding context to promote change. Brit. J. Soc. Work 42, 1132–1150. 10.1093/bjsw/bcs072

[B34] Rivett M. Street E. (2009). Family Therapy in Focus. London: Sage Publications.

[B35] Rustin M. (2015). Psychotherapy in a neoliberal world. Euro. J. Psychother. Counsell. 17, 225–239. 10.1080/13642537.2015.1059869

[B36] Statens helsetilsyn (2001). ”Kvalitetsforbedring i psykisk helsevern. Prosessforbedring i klinisk virksomhet, nr. 5”.).

[B37] Stivale C. J. (2014). Gilles Deleuze: key concepts. Routledge (Chesham: Acumen Publishing Limited).

[B38] Sundet R. (2001). Forskjell og ulikhet–om realitetens makt og maktens realitet. i: Fokus på familien (4).

[B39] Sundet R. (2009). Therapeutic collaboration and formalized feedback: using perspectives from Vygotsky and Bakhtin to shed light on practices in a family therapy unit. Clinic. Child Psychol. Psychiatry 15, 81–95. 10.1177/1359104509341449 19914938

[B40] Sundet R. (2011). Collaboration: Family and therapist perspectives of helpful therapy. J. Marital Fam. Therap. 37, 236–249. 10.1111/j.1752-0606.2009.00157.x 21457287

[B41] Sundet R. Kim H. S. Karlsson B. E. Borg M. Sælør K. T. Ness O. (2020a). A heuristic model for collaborative practice–Part 1: a meta-synthesis of empirical findings on collaborative strategies in community mental health and substance abuse practice. Int. J. Mental Health Syst. 14, 1–16. 10.1186/s13033-020-00376-5 PMC7285576 32528553

[B42] Sundet R. Kim H. S. Karlsson B. E. Borg M. Sælør K. T. Ness O. (2020b). A heuristic model for collaborative practice—part 2: development of the collaborative, dialogue-based clinical practice model for community mental health and substance abuse care. Int. J. Mental Health Syst. 14, 1–12. 10.1186/s13033-020-00377-4 PMC7285597 32528554

[B43] Tilden T. Wampold B. E. (2017). Routine Outcome Monitoring in Couple and Family Therapy. The Empirically Informed Therapist. Cham, Switzerland: Springer.

[B44] Vike H. (2017). Politics and Bureaucracy in the Norwegian Welfare State: An Anthropological Approach. New York, NY: Springer.

[B45] Wampold B. E. Imel Z. E. (2015). The Great Psychotherapy Debate: The Evidence for What Makes Psychotherapy Work. London: Routledge. 10.4324/9780203582015

[B46] White S. Gibson M. (2019). Reassessing Attachment theOry in Child Welfare: A Critical Appraisal. Bristol, VA: Policy Press. 10.1332/policypress/9781447336914.001.0001

[B47] Wilden A. (1987). Man and Woman, War and Peace: The Strategist's Companion. London: Routledge.

[B48] Wise J. M. (2005). “Assemblage,” in Gilles Deleuze. Key Concepts., ed Stivale C.J. (Chesham: Acumen), 77–87.

